# Temporal TCR dynamics and epitope diversity mark recovery in severe COVID-19 patients

**DOI:** 10.3389/fimmu.2025.1582949

**Published:** 2025-07-10

**Authors:** Kriti Khare, Sunita Yadav, Sayanti Halder, Yogiraj Ray, Dipyaman Ganguly, Rajesh Pandey

**Affiliations:** ^1^ Division of Immunology and Infectious Disease Biology, INtegrative GENomics of HOst-PathogEn (INGEN-HOPE) Laboratory, Council of Scientific and Industrial Research (CSIR)-Institute of Genomics and Integrative Biology (CSIR-IGIB), Delhi, India; ^2^ Academy of Scientific and Innovative Research (AcSIR), Ghaziabad, India; ^3^ Infectious Disease and Beliaghata General Hospital, Kolkata, India; ^4^ Department of Infectious Diseases, Shambhunath Pandit Hospital, Institute of Postgraduate Medical Education and Research, Kolkata, India; ^5^ Department of Biology, Ashoka University, Sonipat, Haryana, India; ^6^ IICB-Translational Research Unit of Excellence, CSIR-Indian Institute of Chemical Biology, Kolkata, India

**Keywords:** COVID-19, ICU-admitted patients, TCR dynamics, TCR clonotypes, adaptive regulation

## Abstract

**Introduction:**

Severe COVID-19 is characterized by immune dysregulation, with T cells playing a central role in disease progression and recovery. However, the longitudinal dynamics of the T cell receptor (TCR) repertoire during the course of severe illness remain unclear.

**Methods:**

To investigate temporal changes in adaptive immunity, we analyzed peripheral blood samples from the ICU-admitted severe COVID-19 patients (n = 36) collected at three time points: Day 1 (T1), Day 4 (T2), and Day 7 (T3). Bulk RNA-sequencing was performed to extract TCR repertoires, and cytokine profiles were assessed in parallel. TCR clonotypes were annotated using VDJdb and TCRex to infer potential epitope specificities.

**Results:**

By T3, we observed a 2.3-fold expansion in TCR clonotypes along with increased TCR-β (TRB) chain usage, indicating the emergence of a broad polyclonal T cell response. In contrast, TCR-γ (TRG) chain prevalence declined. Pro-inflammatory cytokines, including IL-1β and IL-6, were reduced over time, marking a shift toward immune resolution. Changes in CDR3 motifs and preferential TRBV gene segment usage were detected, suggesting repertoire adaptation. Additionally, annotated TCR clonotypes at T3 mapped to SARS-CoV-2 and other pathogen-associated epitopes (e.g., CMV, Plasmodium), reflecting possible cross-reactivity or memory T cell recruitment.

**Discussion:**

These findings suggest a coordinated transition from immune dysfunction to recovery in severe COVID-19, marked by expanding TCR diversity, reduced inflammation, and predicted broadening of antigen recognition. The integrated analysis of TCR repertoire dynamics and cytokine profiles provides insights into the adaptive immune mechanisms underlying viral clearance and immune stabilization.

## Introduction

COVID-19, caused by the SARS-CoV-2 virus, has led to a global pandemic, with severe cases often requiring intensive care unit (ICU) admission (1). The immune response to SARS-CoV-2 plays a pivotal role in determining the outcome of infection, especially in severe cases. While some patients recover rapidly, others experience prolonged illness, severe respiratory complications such as acute respiratory distress syndrome (ARDS), and immune dysregulation (2–4). Understanding the dynamics of immune responses, particularly T cell responses, is crucial in deciphering the mechanisms behind disease severity and recovery.

The immune system’s ability to recognize and respond to pathogens is fundamentally shaped by the diversity of the T cell receptors (TCRs), which mediate the recognition of specific antigenic peptides presented by the MHC molecules (5, 6). TCR diversity, generated through V(D)J recombination, encompassing both the clonal expansion of specific TCRs and the overall breadth of the TCR repertoire is critical for mounting an effective immune response against infectious agents (7). In viral infections such as CMV (cytomegalovirus), Influenza, and HIV (human immunodeficiency virus), increased TCR diversity has been shown to correlate with immune recovery, pathogen clearance, and the establishment of long-term immune memory (8–10). Another study has reported where the progression of AIDS is associated with a decrease in TCR diversity, thus providing the importance of TCR repertoire in defining the disease outcomes (11).

Recent studies have demonstrated that viral infections, including SARS-CoV-2 and Dengue virus, can lead to marked alterations in the TCR repertoire, particularly in response to the virus’s diverse epitopes (12–15). In severe cases of COVID-19, the immune system exhibits an initially intense, inflammatory T cell response that can evolve over time, with shifts in clonotype diversity, TCR chain usage, and antigen specificity (16). These dynamic alterations in the TCR repertoire highlight the immune system’s evolving strategies to adapt during diseases, which may provide key insights into both the progression and resolution of the disease. Therefore, elucidating the TCR dynamics at the temporal scale is critical, especially in relation to disease progression and recovery. By tracking TCR clonotypes, diversity, and antigen specificity across multiple time points, more crucial insights can be understood into how the immune system adapts and responds to sustained viral challenges. Previous studies have explored TCR repertoire shifts in various viral infections (17, 18), but the detailed longitudinal analysis of TCR dynamics over the course of COVID-19 infection, particularly in severe cases, remains less well understood.

Given the critical role of TCR-mediated immune responses in controlling viral infections, this study aims to characterize the dynamic changes in the TCR repertoire of the ICU-admitted patients with severe COVID-19. Specifically, we aim to track the evolution of TCR clonotypes, assess their diversity, and explore/elucidate associations with inflammatory cytokine levels and immune cell abundance across three key time points during the course of infection and hospitalization. By integrating repertoire and cytokine data, we seek to understand how adaptive immune responses shift during recovery from severe disease.

To achieve this, we performed total RNA-seq of peripheral blood samples from 36 patients across three longitudinal time points. Following standard pre-processing steps including data quality assessment, adapter trimming, alignment, and assembly of human-mapped reads, we extracted TCR repertoire information. Using public databases such as VDJdb and TCRex, we annotated identified clonotypes to infer potential epitope specificities, including those related to SARS-CoV-2 and other pathogens. This approach enables a comprehensive view of TCR repertoire diversity, potential antigen targeting, and its immunological context during severe COVID-19.

## Material and methods

### Sample collection and patient classification

This study investigated TCR dynamics in severe COVID-19 patients, focusing on longitudinal changes across three time points. The study was part of a randomized controlled trial (RCT) on convalescent plasma therapy (CPT) conducted at Infectious Diseases & Beleghata General Hospital (ID & BG Hospital), Kolkata, India, between May 31 and October 12, 2020 (19, 20). All patient samples used in this study were collected during this period which was the first wave of the COVID-19 pandemic in India. This period preceded both the emergence of SARS-CoV-2 variants of concern and the availability of COVID-19 vaccines; therefore, none of the patients were vaccinated. The RCT was approved by the Central Drugs Standard Control Organisation (CDSCO) (approval no. CT/BP/09/2020) and registered with the Clinical Trial Registry of India (CTRI/2020/05/025209). Ethical approvals were obtained from the Institutional Review Boards of CSIR-Indian Institute of Chemical Biology, Kolkata (IICB/IRB/2020/3 P), Medical College Hospital, Kolkata (MC/KOL/IEC/NON-SPON/710/04/2020), and ID & BG Hospital (IDBGH/Ethics/2429). The study followed the Declaration of Helsinki, with written informed consent obtained from all participants.

Patients were recruited based on RT-PCR-confirmed SARS-CoV-2 infection and severe COVID-19 as per Indian Council of Medical Research (ICMR) guidelines. Inclusion criteria included respiratory distress (respiratory rate >30 breaths/min), SpO2 <90% on room air, and ARDS severity classified as mild (PaO2/FiO2: 200–300 mmHg) or moderate (PaO2/FiO2: 100–200 mmHg) without requiring mechanical ventilation. Pregnant or breastfeeding women, patients under 18 years of age, and individuals with contraindications to blood product transfusion were excluded. A total of 80 patients were randomized into two arms: the standard-of-care (SOC) group (n = 40) receiving guideline-recommended therapy and the CPT group (n = 40) receiving SOC with two doses of ABO-matched 200 ml convalescent plasma on consecutive days (20). Peripheral blood samples (≥5 ml) were collected in EDTA tubes at three time points: day 1 (T1), day 4 (T2), and day 7 (T3) after enrollment. Plasma was isolated via centrifugation at 2,000 g for 10 minutes at 4°C. Clinical data, including comorbidities (e.g., diabetes and hypertension) and medications such as remdesivir, ivermectin, and corticosteroids (dexamethasone and hydrocortisone), were documented. IL-6 receptor blockers and baricitinib were unavailable during the study period. Of the patients, only five received CPT, while the remaining patients in the SOC arm received supportive care.

### RNA Library preparation and high-throughput sequencing

To generate sequencing libraries, we used the Illumina TruSeq Stranded Total RNA Library Prep Gold kit (cat. no. 20020599), following the manufacturer’s guidelines (reference guide: 1000000040499 v00). A total of 250 ng of total RNA, extracted from peripheral blood mononuclear cells (PBMCs) collected from patients at three time points, was used for library preparation. To remove cytoplasmic and mitochondrial ribosomal RNA (rRNA), we utilized target-specific biotinylated oligonucleotides and Ribo-Zero rRNA removal beads. This step is enriched for the non-ribosomal RNA fraction, including mRNA and noncoding RNA species. The resulting RNA was fragmented into smaller fragments using a divalent cation solution at an elevated temperature to ensure uniform and optimal fragment size suitable for library preparation. Fragmented RNA was reverse transcribed into complementary DNA (cDNA) using random primers and SuperScript IV reverse transcriptase. The RNA strand in the RNA-cDNA hybrid was degraded enzymatically using RNaseH, allowing for the synthesis of the second cDNA strand. DNA polymerase I was employed to synthesize the second strand, producing double-stranded cDNA. The double-stranded cDNA was end-repaired to produce blunt ends, followed by adenylation at the 3’ ends. This facilitated the ligation of indexed sequencing adapters, which allow for multiplexing of multiple samples during sequencing. The adapters contained unique indices for each sample, enabling sample identification post-sequencing. To purify the library and remove residual reagents or fragments, AMPure XP beads (Beckman Coulter, cat. no. A63881) were used with a sample-to-bead ratio of 1:1. The purified libraries were assessed for quality and fragment size distribution using the Agilent 2100 Bioanalyzer. Libraries with the expected size distribution were selected for sequencing. The final libraries were sequenced on the Illumina NovaSeq 6000 platform using NovaSeq S2 v1.5 reagents. Sequencing was conducted with a paired end read length of 2 × 101 bp at a loading concentration of 450 pM. This setup ensured high sequencing depth and quality, necessary for comprehensive transcriptomic analysis.

### Pre-processing and analysis of raw sequencing data

For TCR repertoire analysis, transcriptome sequencing data in FASTQ format from COVID-19 patient samples were processed to identify and quantify TCR clonotypes. The raw sequencing reads underwent an initial quality assessment using FastQC (21), which flagged low-quality reads (phred score <20) and adapter contamination. These suboptimal sequences were trimmed using Trimmomatic v0.39 (22), ensuring the retention of only high-quality reads for downstream analysis. TCR clonotypes were identified using MiXCR (23), which aligned reads to V, D, J, and C gene segments based on the IMGT (international ImMunoGeneTics) database. The pipeline assembled these reads to extract the CDR3 regions, essential for characterizing TCR diversity. To enable comprehensive downstream analysis, the MiXCR output was reformatted using VDJtools (24), a versatile toolkit for analyzing TCR data. This step standardized the data for further exploration of TCR diversity, clonotype frequency distributions, and repertoire dynamics across longitudinal samples.

### CDR3 sequence and TCR diversity analysis

We conducted an in-depth analysis of CDR3 sequences using VDJtools, applying default parameters to evaluate multiple features of the TCR repertoire. This included basic statistical summaries, length distribution of CDR3 regions, patterns of V(D)J segment usage, and motif identification within amino acid sequences. To assess clonal diversity, we calculated a range of metrics, such as Chao1, D50, Shannon-Wiener, and Inverse Simpson indices, which collectively provided information on the richness, abundance, and evenness in the TCR clonotypes. TCR repertoire diversity refers to the number and distribution of unique clonotypes, commonly measured by indices such as Shannon or Chao1. Clonality describes the degree of expansion or dominance of specific clones, and is inversely related to diversity. Heterogeneity broadly describes variability within the repertoire and is used here in a descriptive sense. Significance for clonotypes and diversity parameters across time points was conducted using the Wilcoxon signed-rank test. Furthermore, the distribution of TCR chains—TRA (α), TRB (β), TRD (δ), and TRG (γ)—was analyzed by calculating their relative abundances within severity groups. Statistical significance in chain usage patterns across groups was assessed using the Fisher exact test, providing insights into the variability and preferential selection of specific TCR chains in ICU-admitted severe COVID-19 patients.

### Gene usage analysis with principal component analysis

To investigate the differences in TCR gene usage across dengue severity subgroups, we performed a comparative analysis of V, J, and VJ segment usage based on their read frequencies within each sample. Principal component analysis (PCA) was employed to reduce the complexity of high-dimensional TCR gene usage data and identify patterns of variation across time. By projecting the V segment usage into principal components (PCs), we could visualize and compare how the usage of specific segments evolves over time. This approach helped identify key segments that contribute significantly to the variation in TCR repertoire and track the temporal shifts in TCR diversity. Additionally, the statistical significance of differences in gene usage was assessed using the Wilcoxon signed-rank test.

### CDR3 motif and amino acid composition analysis

To identify and analyze unique patterns and conserved domains associated with TCR diversity and specificity, CDR3 motif analysis was conducted. Sequence logos were generated using the WebLogo tool (25) to visualize the positional frequency of amino acids within CDR3 sequences, highlighting conserved motifs and key residues critical for antigen recognition. Additionally, the amino acid composition of CDR3 regions was assessed by calculating the relative frequencies of hydrophobic, polar, and charged residues, providing insights into their biochemical properties. Statistical comparisons were performed to identify significant differences in amino acid distributions across groups or time points using Kruskal-Wallis with Dunn’s comparison test.

### Quantification of plasma cytokines

Cytokine levels in plasma samples collected in EDTA vials were measured using the Bio-Plex Pro Human Cytokine Screening Panel (48-Plex Assay, Bio-Rad). This multiplex assay enabled the simultaneous detection and quantification of 48 cytokines, including FGF basic, eotaxin, G-CSF, GM-CSF, IFN-γ, IL-1β, IL-1ra, IL-1α, IL-2Rα, IL-3, IL-12 (p40), IL-16, IL-2, IL-4, IL-5, IL-6, IL-7, IL-8, IL-9, GRO-α, hepatocyte growth factor, IFN-α2, leukemia inhibitory factor, MCP-3, IL-10, IL-12 (p70), IL-13, IL-15, IL-17A, IL-18, IP-10, MCP-1, MIG, NGF-β, SCF, SCGF-β, SDF-1α, MIP-1α, MIP-1β, PDGF-BB, RANTES, TNF-α, TNF-β, VEGF, CTACK, MIF, TRAIL, and M-CSF. Plasma samples were diluted 1:3 with the assay-specific diluent and processed according to the manufacturer’s protocol. The analysis was performed on a Bio-Plex 200 System (Bio-Rad) to generate cytokine concentration data (pg/ml) for each sample.

### Immune cell subset quantification

Following plasma separation, whole blood samples were treated with RBC lysis buffer to remove erythrocytes. The resulting leukocyte pellet was fixed with 1% paraformaldehyde and stained with fluorochrome-conjugated antibodies (BD Biosciences) targeting specific surface markers for immune cell subset identification. Stained samples were acquired using a FACSAria III flow cytometer, and data analysis was performed using FlowJo software to quantify the various immune cell populations.

### TCR clonotype annotation and epitope prediction

To annotate TCR clonotypes and predict their epitope specificities in COVID-19 and other infectious diseases, we employed two publicly available resources, VDJdb (26) and TCRex (27). VDJdb is a comprehensive repository comprising experimentally validated T-cell receptor sequences and their associated antigen specificities. This resource enables us to identify TCRs capable of recognizing specific epitopes presented within defined MHC contexts. For epitope binding predictions, we utilized TCRex, a tool designed to analyze the TCR beta chain (TRB-β). TCRex evaluates the complementarity-determining region 3 (CDR3) amino acid sequences and corresponding V/J gene usage, employing random forest classifiers. These classifiers are trained on epitope-specific TCR data derived from several well-established databases, such as McPAS-TCR (28), the VDJ database (VDJdb), and the ImmuneCODE database (29). TCR–epitope associations were inferred using VDJdb and TCRex, both of which match input TCR CDR3 sequences and V/J gene usage against curated databases of experimentally validated TCR–peptide–MHC interactions. Only exact or high-confidence matches were retained for epitope assignment. While these tools provide valuable insights into the antigen recognition potential of TCR clonotypes, it is important to note that the resulting annotations are based on in silico predictions. These predictions rely on existing public TCR–epitope associations and are inherently limited by database representation, HLA restriction context, and lack of experimental validation. Therefore, all reported epitope matches, particularly those related to CMV, EBV (Epstein-Barr virus), and Plasmodium, should be interpreted as computationally inferred and not as direct evidence of functional or cross-reactive antigen recognition. By integrating these resources, we gained valuable insights into the antigen recognition potential of TCR clonotypes, providing a deeper understanding of immune responses in the context of infectious diseases.

### Quantification and statistical analysis

Statistical analyses were conducted using non-parametric tests, including the Repeated Measure ANOVA test, the Wilcoxon signed-rank test, Mann-Whitney U test, Kruskal-Wallis test, and Fisher’s exact test, to account for the distribution of the data. These analyses were performed using GraphPad Prism 9 (licensed version). Data visualization was achieved through an integrative approach, utilizing tools such as VDJtools for TCR repertoire analysis, RAWGraphs (30) for graphical representation, SRplots (31) for additional data interpretation, WebLogo for motif visualization, and R (version 4.3.2) for advanced statistical graphics. Statistical significance was set at *p* < 0.05 across all tests, ensuring robust interpretation of the findings.

## Results

### Clinical demographics and patient classification

**Figure 1:**
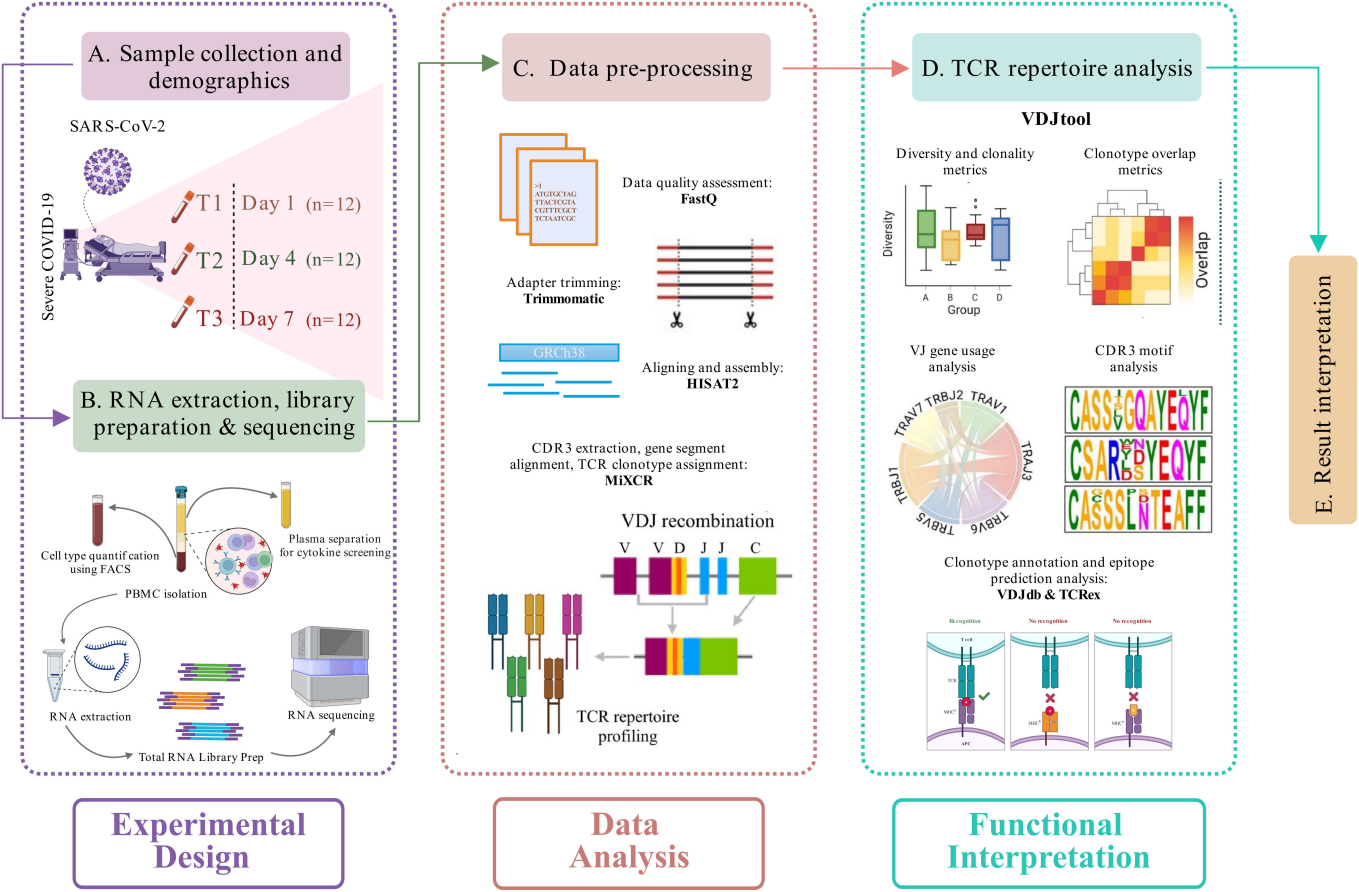
Study design and experimental workflow. **(A)** Blood sample collection and demographics of the longitudinally severe COVID-19 patients recruited in this study. **(B)** RNA isolation, followed by library preparation and sequencing of the samples. A cytokine screening and cell type quantification were performed using plasma and blood samples, respectively, of each patient. **(C)** Pre-processing of the sequencing data, including data quality assessment, adapter trimming, alignment, and assembly of human-mapped reads, followed by TCR clonotype identification (MiXCR). **(D)** TCR repertoire downstream analysis (VDJtool), including clonality and diversity metrics, clonotype overlap metrics, VJ usage, and CDR3 motif analysis, followed by disease-specific clonotype annotation and epitope prediction (VDJdb and TCRex). **(E)** Result interpretation.

This study involved the collection of longitudinal blood samples from the COVID-19 patients admitted to the intensive care unit (ICU) at the ID and BG Hospital in Kolkata, India. The study design, illustrated in **Figure 1**, outlines the workflow: sample collection, library preparation, sequencing, data pre-processing, and downstream TCR repertoire analysis. Blood was drawn at three distinct time points: upon admission (Day 1, T1), four days after admission (Day 4, T2), and seven days post-initial collection (Day 7, T3). All patients included in the study were classified as severe COVID-19 cases based on the guidelines provided by the Indian Council of Medical Research (ICMR). Clinical evaluation revealed that these patients suffered from acute respiratory distress syndrome (ARDS), as evidenced by their SpO2/FiO2 ratio, which ranged between 100 and 300 mmHg, indicating compromised oxygenation. The treatment protocols varied among patients but commonly included the use of corticosteroids such as hydrocortisone and dexamethasone, antiviral drugs like remdesivir and ivermectin, alongside other supportive medications. A subset of the cohort (five patients) also received convalescent plasma therapy (CPT) as part of their treatment plan. Furthermore, prevalent comorbidities such as type 2 diabetes and hypertension were frequently observed, adding to the complexity of their clinical profiles (**Supplementary Table S1**).

### Temporal dynamics of TCR repertoires in ICU-admitted severe COVID-19

**Figure 2:**
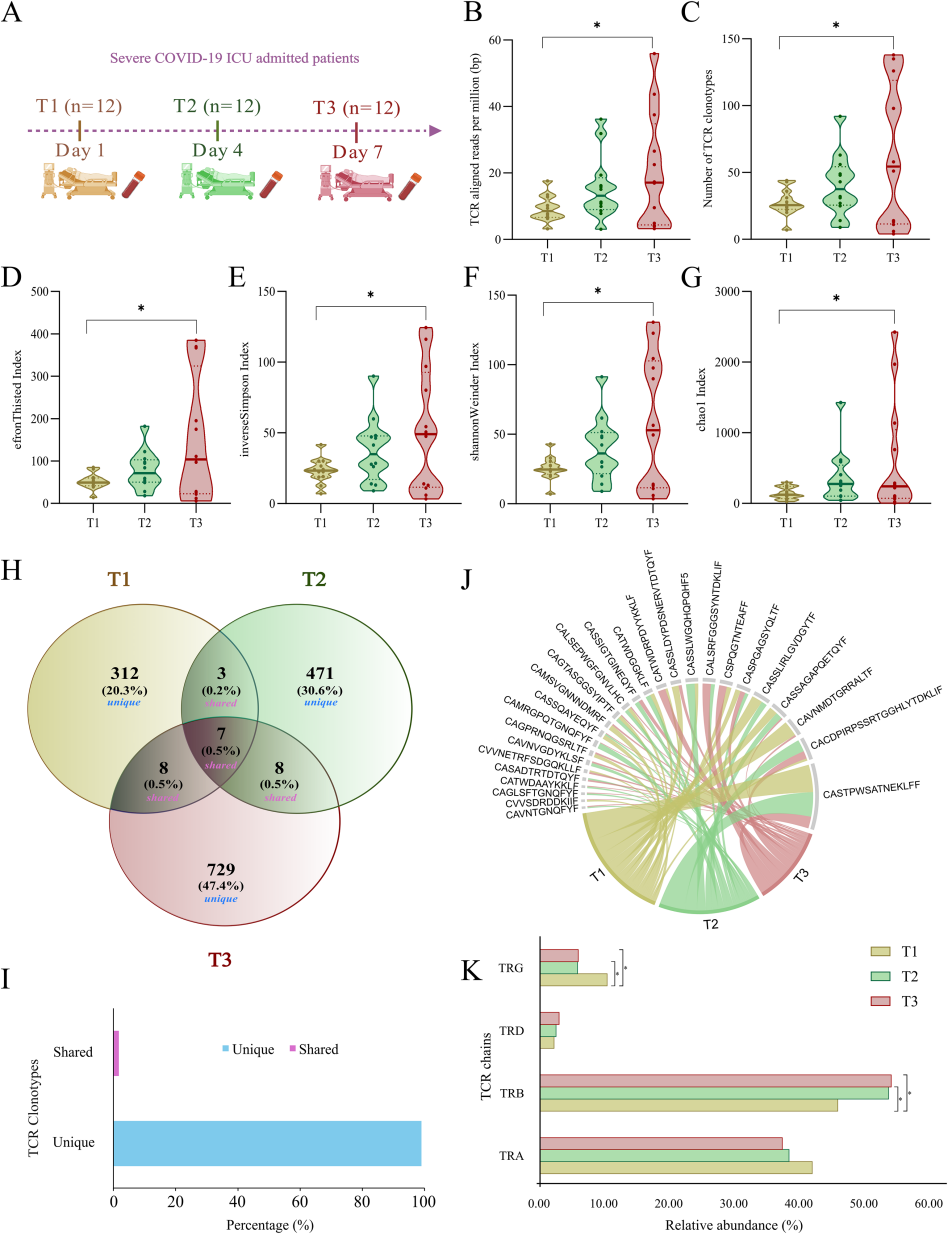
Sample distribution and TCR clonotype dynamics in ICU-admitted severe COVID-19 patients across T1, T2, and T3. **(A)** Blood collection of ICU-admitted severe COVID-19 patients at three time-points (T1, T2, and T3). **(B, C)** Violin plots depict the distribution of **(B)** TCR-aligned reads per million (bp) and **(C)** number of TCR clonotypes across T1, T2, and T3. **(D-G)** Violin plots representing significant differences within TCR clonal diversity indices, including **(D)** EfronThisted index, **(E)** inverse Simpson index, **(F)** Shannon Weiner index, and **(G)** chao1 index across three time points of severe COVID-19 patients. **(H)** Venn diagram represents the number and percentage of unique and public (shared) TCR clonotypes between T1, T2, and T3. **(I)** The bar plot depicts the overall percentage of unique (~99%) and shared (~1%) TCR clonotypes across all the samples. **(J)** Chord diagram depicting shared (public) TCR clonotypes between either two or three time points among severe COVID-19 patients. **(K)** Bar plot shows relative abundance differences between four chains of TCR, including TRA (α), TRB (β), TRD (δ), and TRG (γ) across T1, T2, and T3. Statistical significance for TCR-aligned reads, clonotypes, and diversity indices was calculated using a repeated measure ANOVA test (p < 0.05). Fisher’s exact test was performed for calculating the statistical significance (p < 0.05) between TCR chains across T1, T2, and T3. Data are represented as median +/- SEM. The significance value is denoted as ∗, where ∗ indicates p ≤ 0.05, ∗∗ indicates p ≤ 0.01, ∗∗∗ indicates p ≤ 0.001, and ∗∗∗∗ indicates p ≤ 0.0001.

A total of 36 samples were included in this study, with 12 samples collected at each time point: T1 (Day 1), T2 (Day 4), and T3 (Day 7) (**Figure 2A**). To gain insights into the dynamics of TCR repertoire during severe COVID-19, we employed MiXCR and VDJtool for bulk TCR repertoire analysis across the three time points. **Figure 2B** illustrates a significant differential distribution of aligned TCR reads per million sequencing reads between T1 and T3 (**Supplementary Table S2**). Analysis identified a total of 1571 unique TCR clonotypes, with 330, 489, and 752 clonotypes detected at T1, T2, and T3 time-points, respectively. The details of median and average clonotype counts for these longitudinal groups are present in **Supplementary Table S3**. Notably, there was a marked increase in the number of TCR clonotypes at T3 compared to T1 (**Figure 2C**), indicating a shift in the TCR landscape as the immune response evolved. Additionally, TCR diversity was significantly higher at T3 than at T1 (**Figures 2D–G**) (**Supplementary Table S4**), further suggesting an expansion of the TCR repertoire. The increased clonotype count and diversity at T3 possibly suggest robust clonal expansion, could be influenced by persistent viral antigens that stimulate the immune system to recruit a wider range of TCRs, targeting multiple viral epitopes.

### Enhanced TCR heterogeneity and elevated TCR-β chain usage at T3 in ICU-admitted severe COVID-19

The extremely diverse repertoire of TCR sequences allows the immune system to develop a specific response to almost any possible pathogen. This diversity is generated through a V(D)J somatic recombination event. Due to an enormous TCR heterogeneity in an individual, the probability of shared clonotypes is very low. In this study, we investigated the presence of both unique and shared clonotypes across three time points of the same individuals, and we discovered that ~99% of the TCR clonotypes were unique across each time point, with only a few shared between either T2 or T3 time-points (**Figures 2H–J**). The distribution of unique and shared clonotypes across the cohort further highlighted a high degree of clonal turnover over time, supporting the dynamic nature of the TCR repertoire during the course of severe COVID-19 (**Supplementary Table S9**).

With four different chains, the TCR repertoire is dynamic in eliciting immune responses. The presence of α (TRA) and β (TRB) chains constitutes around 95% of the T cell receptor and responds to the pathogen in an antigen-specific manner to induce cytokine productions followed by B cell maturation and antibody secretion. However, a small fraction of T cells carrying γ (TRG) and δ (TRD) receptor chains are essential in the initial immune and inflammatory responses. In our data, ~90% of the TCR clonotypes consist of α (TRA) and β (TRB) chains, while ~10% of them belong to γ (TRG) and δ (TRD) chains. The most dominant TCR chain was β (TRB) in the COVID-19 patients. Interestingly, the β (TRB) chain was significantly increased in T3 compared with T1 and T2 (**Supplementary Table S5**). However, the γ (TRG) chain was significantly decreased in T3 compared to T1 (**Figure 2K**). This suggests the involvement of an active T cell response and reduced inflammatory response with prolonged COVID-19 disease severity.

### Dynamic trend of V and J segment usage between T1 and T3 time-points

**Figure 3:**
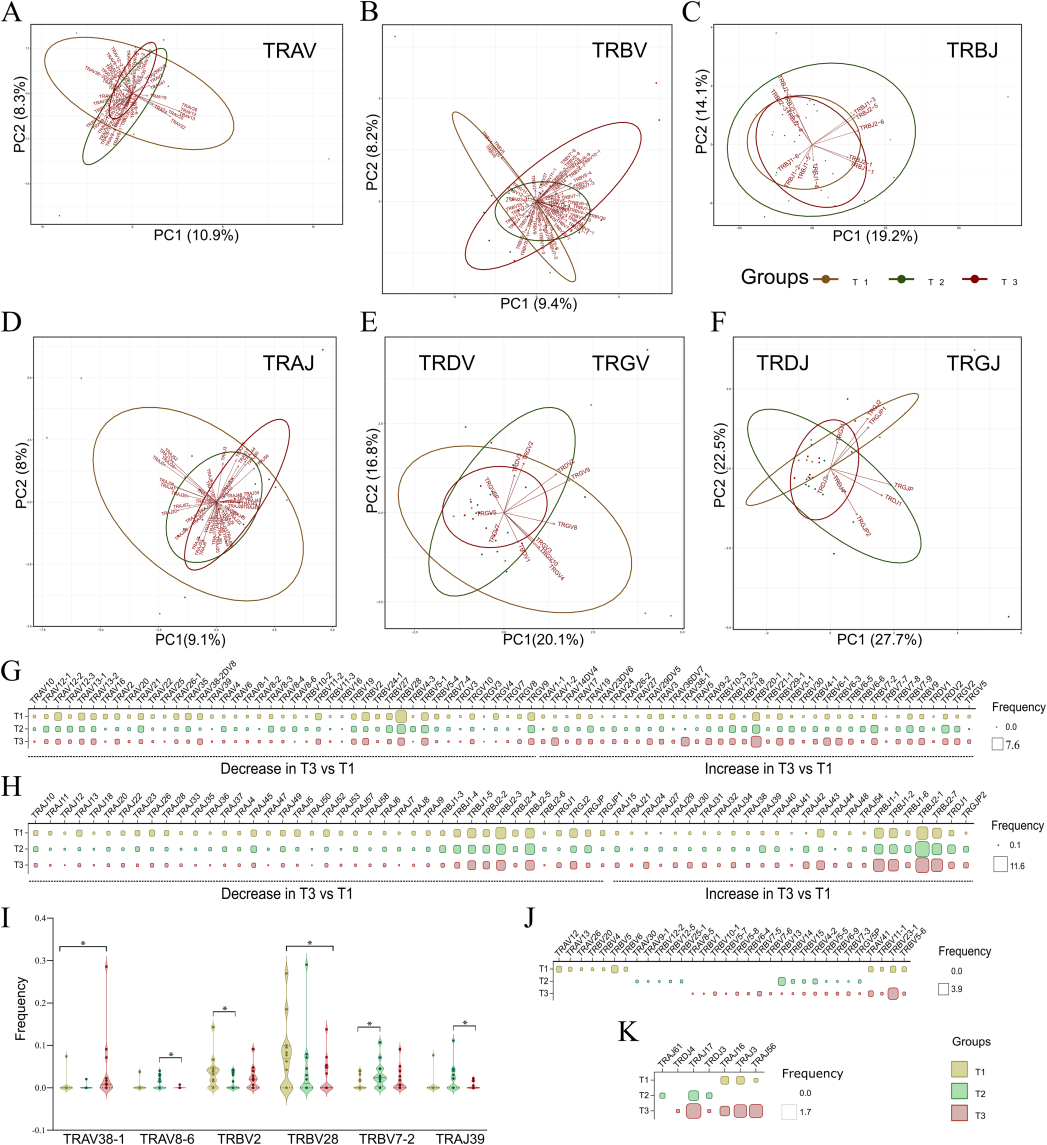
Principal component analysis (PCA) plots with dynamic V and J segment usage across T1, T2, and T3. **(A-F)** Principal component analysis (PCA) plots depict variance among shared V and J segments for each TCR chain, including **(A)** TRAV, **(B)** TRBV, **(C)** TRBJ, and **(D)** TRAJ; **(E, F)** Combined PCA plots for **(E)** TRDV and TRGV, and **(F)** TRDJ and TRGJ, across T1, T2, and T3. **(G, H)** The matrix plot shows the frequency distribution in shared **(G)** V and **(H)** J segment usage across time points, illustrating either an increase or decrease in their usage from T1 to T3. **(I)** Violin plot represents significant common V and J segments across the three time points. **(J, K)** The matrix plot shows the frequency distribution in unique **(J)** V and **(K)** J segments across the three time points. Wilcoxon’s signed rank test was performed to calculate significance for shared V and J segments across T1, T2, and T3. Data are represented as median +/- SEM. The significance value is denoted as ∗, where ∗ indicates p ≤ 0.05, ∗∗ indicates p ≤ 0.01, ∗∗∗ indicates p ≤ 0.001, and ∗∗∗∗ indicates p ≤ 0.0001.

During the V(D)J recombination event, regulatory factors such as chromatin accessibility, recombination signal sequence efficiency, and the spatial organization of gene segments guide the joining of different V and J segments (32). This generates a diverse TCR repertoire that is ready to respond to possibly any pathogen encountering the host. Therefore, upon an infection, the V and J segments, through their preferential or biased usage, play a crucial role in defining the repertoire dynamics. Utilizing the bulk TCR data for three time points, we identified a total of 112 V and 72 J segments within the TCR repertoire, of which 80 V and 65 J were shared across all the three time-points (**Supplementary Figure S1**). **Figures 3A–F** represents the principal component analysis (PCA) plots for distribution of V and J segments shared across T1, T2, and T3. From the PCA plots, it can be seen that the V and J segments do not show large variance as the points representing the segments are overlapping across the time points. Interestingly, the TRBV segments (**Figure 3B**) across T1 and T3 form separate clusters with minimal overlap, suggesting plausible selective usage with disease progression.

As the clonotypes were significantly different across T1 and T3, we delved deeper to find whether there is a selective bias for V and J segments between the first and third time-points. **Figures 3G, H** depict differential usage of V and J segments, respectively, which either show a decreased or increased trend from T1 to T3. Differential analysis revealed increased usage of TRBV10-3, TRBV20-1, TRBV18, TRBV29-1, TRBV3-1, TRBV4-1, TRBV6-1, TRBV6-3, TRBV6-5, TRBV7-8, and TRBV9 at T3, along with an increased trend in TRBJ1-1, TRBJ1-2, TRBJ1-6, TRBJ2-1, and TRBJ2–7 segments. Conversely, decreased usage was observed in the TRGV segments, such as TRGV10, TRGV3, TRGV4, TRGV7, TRGV8, and TRGV9, and in TRGJ segments like TRGJ1, TRGJ2, TRGJP, and TRGJP1. As the decrease was not large due to low variance observed across PCA plots, only 5 V and one J segment were found to be significantly different (**Figure 3I**) (**Supplementary Table S6**). TRAV38–1 was observed to increase at T3 compared to T1, whereas TRBV28 decreased significantly from T1 to T3.

A few V and J segments were uniquely present or only shared among two time-points. Out of 8 uniquely V segments used at T3, 7 belonged to the TRBV chain, including TRBV1, TRBV10-1, TRBV5-7, TRBV5-8, TRBV6-4, TRBV7-5, and TRBV7-6. We also observed the absence of unique TRAV segments along with a decrease of TRGV5P at T3 (**Figure 3J**). Among J segments, only TRDJ4 was selectively used at the third time point (**Figure 3K**). This reveals the dynamic usage of V and J segments observed between T1, T2, and T3. Among the VJ pairs, we identified a total of 62 VJ pairs common across three time-points (**Supplementary Figure S2**). However, none was found to be significantly different between any of the time points.

### CDR3 length and amino acid motif differentiation indicate adaptive immune changes

**Figure 4:**
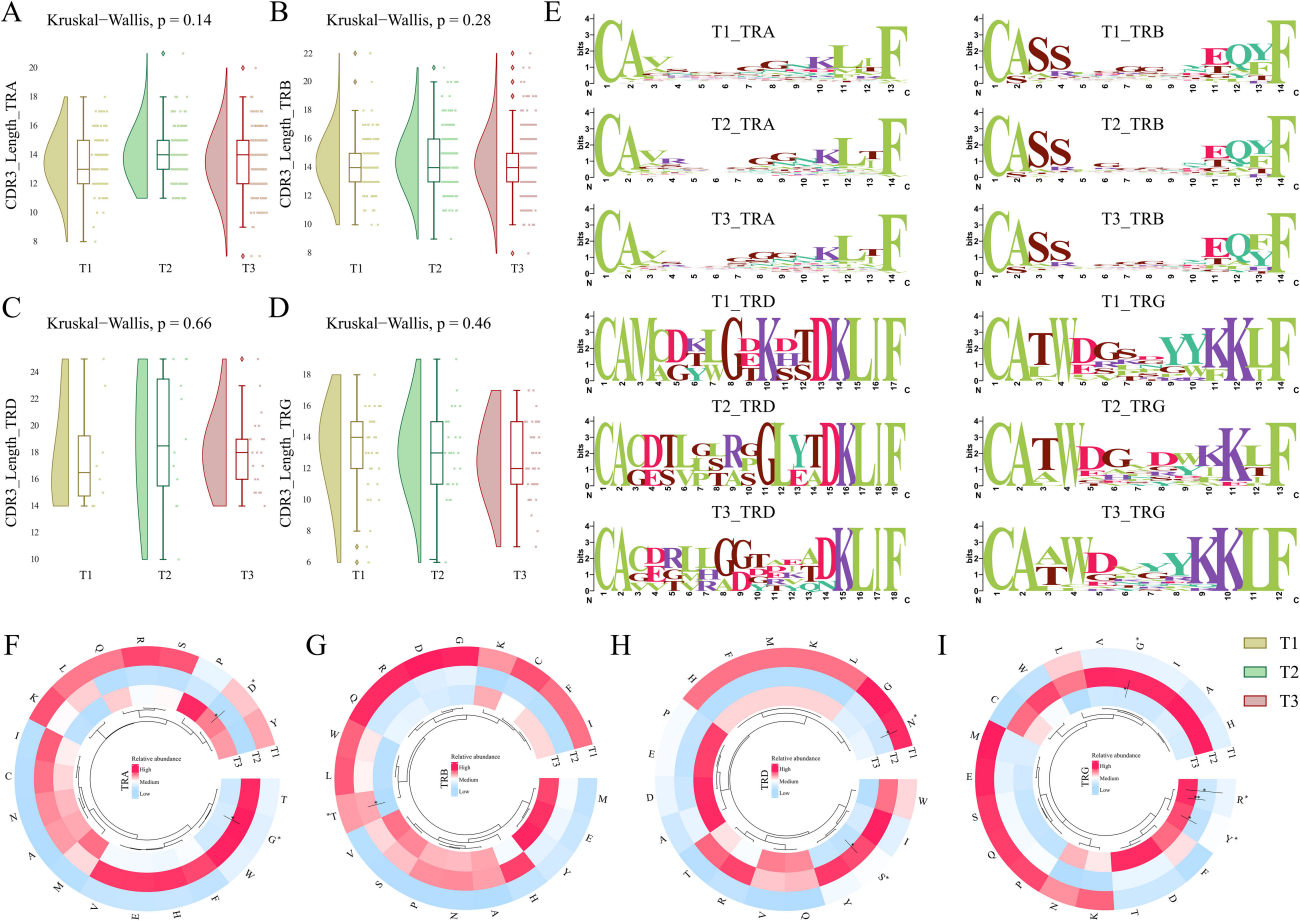
CDR3 length, motif, and amino acid composition across T1, T2, and T3 time points for severe COVID-19. **(A-D)** The raincloud plots represent median CDR3 lengths for each chain, including **(A)** TRA (α), **(B)** TRB (β), **(C)** TRD (δ), and **(D)** TRG (γ) across the three time points. **(E)** Weblogos depicting the CDR3 motifs within median lengths for each chain across T1, T2, and T3. **(F-I)** The circular heatmaps show the relative abundance of individual amino acids for **(F)** TRA (α), **(G)** TRB (β), **(H)** TRD (δ), and **(I)** TRG (γ) chains across three time points in severe COVID-19 patients. A Kruskal-Wallis (p < 0.05) test was performed for calculating significance for CDR3 lengths across T1, T2, and T3. The significance of amino acids between the temporal severe COVID-19 patients was performed using the Man-Whitney U test (p < 0.05). Data are represented as median +/- SEM. The significance value is denoted as ∗, where ∗ indicates p ≤ 0.05, ∗∗ indicates p ≤ 0.01, ∗∗∗ indicates p ≤ 0.001, and ∗∗∗∗ indicates p ≤ 0.0001.

Being the major region of variability in the TCR repertoire, the CDR3 amino acids play a crucial role in antigen recognition, TCR repertoire diversity, clonal expansion, and immune escape mechanisms. Therefore, we looked into the CDR3 amino acid lengths within all the chains of TCR clonotypes across the T1, T2, and T3. α-CDR3 (TRA) sequences had lengths ranging from 8 to 21 amino acids, with a median of 14 amino acids. β-CDR3 (TRB) sequences had lengths ranging from 8 to 22 amino acids, with a median of 14 amino acids. γ-CDR3 (TRG) sequences had lengths ranging from 6 to 19 amino acids, with a median of 13, while δ-CDR3 (TRD) sequences had lengths ranging from 10 to 25 amino acids, although they had a longer median of 18 amino acids. When looked across time-points, the TRG (γ) chain exhibited a decrease in the median length at T3 compared to T1. However, none of the differences observed for CDR3 length was found to be significant across the time-points (Kruskal-Wallis > 0.05) (**Figures 4A–D**). Also, when investigated within a single time point for all the chains, the CDR3 length of TRG (γ) was maximally dropped at the T3 stage (**Supplementary Figure S3**).

The presence of specific motifs within the CDR3 region also impacts the ability and affinity of the TCR clonotype toward antigen response. Among all the CDR3 chains (α (TRA), β (TRB), δ (TRD), and γ (TRG)), a decrease of glycine residues (G) was observed at T3 in the central region of the sequence logo. δ-CDR3 (TRD) exhibited an introduction of valine (V) at positions 3, 4, 6, and 12 along with glutamine (Q) at position 13 of the weblogo in T3 compared to T1 (**Figure 4E**). Interestingly, serine (S) was observed to be reduced from the δ (TRD) and γ-CDR3 (TRG) sequences at T3 when compared to T1. This differentiation of specific motifs across the 7-day time scale of severe COVID-19 indicates the variability and dynamic nature of the CDR3 region in the TCR repertoire.

We also investigated whether there is a substantial difference in the usage of specific amino acids across different chains of TCR between time-points and how they impact the COVID-19 severity. Between the TCR chains, we observed that glycine (G) was mostly used in the TRA (α) and TRB (β). Interestingly, there was a decrease in the usage of glycine at T3 across all the TCR chains. Serine (S) and asparagine (N) levels were high in the TRA (α) chain; the TRB (β) chain was observed to be enriched with serine (S), glutamic acid (E), and proline (P) amino acids; leucine (L), aspartic acid (D), and threonine (T) levels were increased in the TRD (δ); while lysine (K), tryptophan (W), glutamic acid (E), and tyrosine (Y) levels were pronounced in the TRG (γ) chains (**Supplementary Figure S3**). The structural interaction of the CDR3 loops with the antigen is dictated by local physical interactions driven by the physicochemical properties of the amino acids of the loops (33). Therefore, we investigated whether specific amino acids are elevated or reduced in a particular chain with an infection stage time-point. Within, TRA (α) and TRG (γ) chain glycine were significantly reduced at T3 compared to T2. Aspartic acid (D) was observed to be significantly high in the TRA (α) chain at T3 compared to T2. Threonine in TRB (β) decreased significantly at T3 vs. T2. Serine (S) is significantly reduced at T3 compared to T2 in the TRD (δ) chain. In the same chain, we also observed a significant decrease in the level of asparagine (N) at T2 compared to T1. In the TRG (γ) chain, a significant increase in both arginine (R) and tyrosine (Y) was observed at T3 compared to T1 and T2 (**Figures 4F–I**). A significant increase in TRB (β) chain and decreased TRG (γ) chain, together with a differential relative abundance of amino acids at T3 compared to T1, suggests their crucial involvement in antigen recognition and potent immune response during COVID-19.

### Decreased inflammatory cytokines and enhanced immune cell abundance with disease progression

Cytokine profiles and cell abundance play pivotal roles in defining the course of infection and influencing disease outcomes. Elevated pro-inflammatory cytokines and robust T-cell activation typically drive active immune responses; however, their dysregulation, such as during “cytokine storm,” can lead to excessive inflammation, tissue damage, and immune exhaustion, often worsening clinical outcomes.

**Figure 5:**
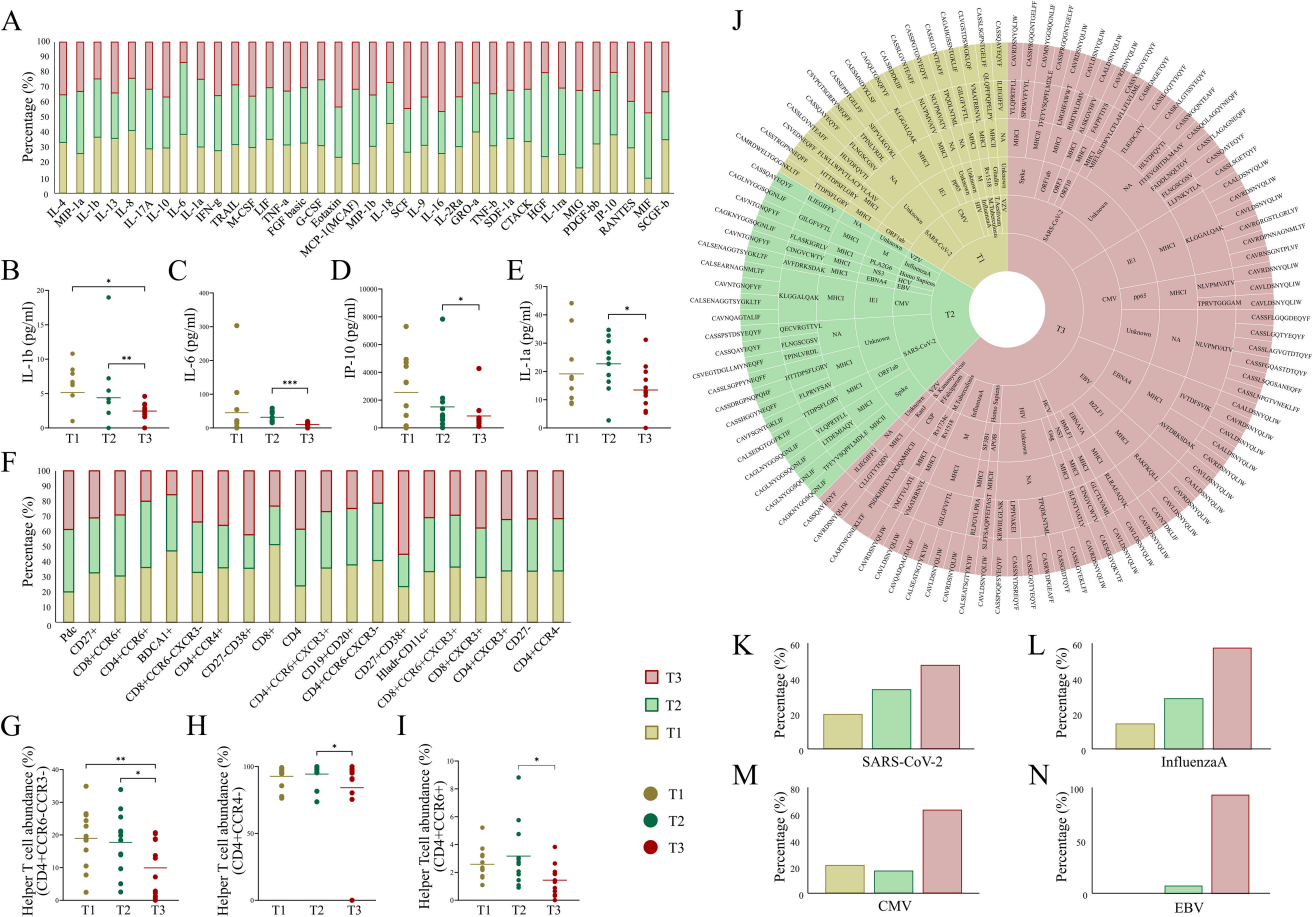
Dynamics of cytokine, cell abundance, and TCR-specific epitopes across ICU-admitted longitudinal COVID-19 cases. **(A)** Bar plot depicts cytokine levels (pg/ml) in severe COVID-19 patients across the three time points. **(B-E)** Aligned dot plots show significant differences for **(B)** IL-1β, **(C)** IL-6, **(D)** IP-10, and **(E)** IL-1α across T1, T2, and T3. **(F)** Bar plot depicts differential abundance of cell types (cell surface markers) in severe COVID-19 patients across three time points. **(G-I)** Aligned dot plots show significant differences for **(G)** CD4+CCD6-CCR3-helper T-cell, **(H)** CD4+CCR4-helper T-cell, and **(I)** CD4+CCR6+ helper T-cell populations across T1, T2, and T3. **(J)** Sunburst plot represents species-specific epitope and MHC interaction with TCR clonotypes across T1, T2, and T3. **(K)** Bar plots show relative abundance of species-specific epitopes, including **(K)** SARS-CoV-2, **(L)** InfluenzaA, **(M)** CMV (Cytomegalovirus), and **(N)** EBV (Epstein-Barr virus) across the three time points in severe COVID-19 patients. Significance was performed using Wilcoxon’s signed rank test (p < 0.05). Data are represented as median +/- SEM. The significance value is denoted as ∗, where ∗ indicates p ≤ 0.05, ∗∗ indicates p ≤ 0.01, ∗∗∗ indicates p ≤ 0.001, and ∗∗∗∗ indicates p ≤ 0.0001.

In this study, plasma cytokine levels and cell abundance were quantified across patients at each time point during their ICU admission (**Supplementary Tables S7**, **S8**) (**Figures 5A, F**). Notably, IL-1β, a key pro-inflammatory cytokine that promotes T-cell activation, showed a significant decrease at T3 compared to T1 and T2 (**Figure 5B**). Likewise, other pro-inflammatory cytokines, including IL-6, IP-10, and IL-1a, exhibited a decreasing trend from T1 to T3, where the decrease from T2 to T3 was found to be significant (**Figures 5C–E**). Similarly, IL-10, an anti-inflammatory cytokine, was observed to be increased at T3 compared to T1 and T2, however it was non-significant. These cytokines play critical roles in T-cell activation, proliferation, and recruitment, processes heavily influenced by MHC-mediated antigen presentation. The decline in pro-inflammatory mediators along with elevated anti-inflammatory activity indicates a marked reduction in inflammation and focusing of specific adaptive response, suggesting a transition toward immune stabilization and potential recovery by the seventh day of SARS-CoV-2 infection (34). Additionally, a significant decrease in the helper T-cell populations at T3 was observed compared to earlier time points (**Figures 5G–I**). This reduction aligns with a shift from an active immune phase to a more regulated state, potentially reflecting resolution of inflammation and preparation for immune memory formation. Together, these findings highlight a dynamic interplay between cytokine levels and T-cell populations, underscoring their critical roles in mediating the trajectory of immune responses and influencing recovery during viral infections.

### Increased diversity of epitopes suggests polyclonality in TCR-specific antigenic response

The immune system’s ability to mount a robust and adaptive response against viral infections relies heavily on the diversity of TCRs capable of recognizing a wide range of pathogen-derived epitopes (8, 9). Polyclonal T cell responses, marked by the activation of multiple TCR clones, are essential for the efficient recognition of antigens and the subsequent immune recovery. Here, we sought to understand the dynamics of TCR clonotypes and their correlation with antigenic diversity and disease outcome in the severe COVID-19 patients at three time-points (T1, T2, and T3) by annotating the TCR repertoire using the VDJdb and TCRex resources.

Our analysis revealed a noteworthy increase in epitope diversity at T3 compared to both T1 and T2. Specifically, at T3, there was a broader selection of epitopes from SARS-CoV-2, including those targeting key viral proteins such as the spike, ORF1ab, and ORF3 proteins, which likely corresponds to MHC class I-mediated antigen presentation (**Figure 5J**). This suggests that over time, a broader repertoire of viral peptides is processed and presented by the MHC class I molecules to cytotoxic CD8+ T cells. This increase in epitope diversity suggests a shift could reflect enhanced viral clearance mechanisms and a more comprehensive immune response through epitope evolution (6, 35). Additionally, the analysis showed the presence of disease-specific epitopes, including those for CMV (Cytomegalovirus), EBV (Epstein-Barr virus), and *Plasmodium falciparum*, which were enriched at T3 (**Figures 5K–N**). This phenomenon likely represents cross-reactivity of TCRs, a feature of the immune response wherein certain TCRs recognize epitopes from different pathogens due to structural similarities (36). Such cross-reactivity may enhance the expansion of diverse TCRs, indicating a polyclonal antigenic response critical for recognizing and clearing residual or variant viral epitopes (7). These findings support the hypothesis that the interplay between MHC-mediated antigen presentation and cytokine dynamics drives a shift from acute inflammation to a more stabilized immune state. The increase in epitope diversity and reduced cytokine levels may signal that MHC-mediated immune recognition is adapting to the pathogen, focusing on effective viral clearance while minimizing inflammatory damage.

In summary, our observations at T3 suggest that the immune system is shifting toward a more diverse and polyclonal response, which is characteristic of the recovery phase of severe COVID-19. This expanded epitope recognition and diverse TCR activation are critical for the immune system’s ability to resolve the infection, clear remaining viral particles, and establish long-term immune memory.

## Discussion

The dynamics of TCR repertoires provide invaluable insight into the immune response during severe COVID-19 infection. While the study cohort was part of a randomized controlled trial investigating convalescent plasma therapy (CPT), only five patients received the intervention. Due to this limited sample size, the impact of CPT on TCR dynamics and expression profiles was not evaluated in this study. By analyzing TCR clonotypes across three distinct time-points (T1, T2, and T3), we observed significant alterations in the diversity, clonality, and specificity of TCR responses. These changes were notably aligned with the progression of the disease, from an acute immune response to a potential resolution phase and offer key insights into the mechanisms underlying immune recovery during viral infections. Our study demonstrated a significant increase in both the number of TCR clonotypes and the overall TCR diversity by T3 compared to T1. At T3, we observed a 2.3-fold increase in clonotype numbers, suggesting an enhanced clonal expansion in response to sustained viral antigenic stimulation. This observation supports previous studies that have shown that prolonged viral infections often lead to the recruitment of a broader pool of TCRs as the immune system seeks to address ongoing pathogen challenges (8). This increase in clonotype diversity is indicative of a polyclonal T cell response, essential for mounting an effective immune defense and resolving the infection.

The concept of polyclonality in viral infections is well established in the context of adaptive immunity. A study suggests that the presence of a diverse pool of TCRs enhances the immune system’s capacity to recognize a wide variety of viral epitopes, which is crucial in controlling infections like COVID-19 (12, 37, 38). The observed increase in TCR diversity at T3 is consistent with this notion, as the immune system adapts to recognize not only the core SARS-CoV-2 epitopes but also possibly residual or variant viral strains, thereby enhancing immune clearance. Our study also reveals significant changes in TCR repertoire dynamics during the progression of severe COVID-19, particularly in the usage of TCR-β (TRB) chains. The increase in TCR-β (TRB) chain usage at T3, compared to T1 and T2, is consistent with findings from other viral infections where TCR-β (TRB) expansion is linked to an adaptive immune response (39). The β (TRB) chain, which plays a crucial role in antigen recognition, cytokine production, and B cell activation, expands as T cells engage with viral epitopes. This increase in TCR-β (TRB) usage at T3 suggests an ongoing adaptive immune response aimed at resolving the infection (40). Previous studies have shown that in persistent infections, such as CMV and HIV, the expansion of (TRB) TCR-β-bearing T cell populations is critical for viral control and recovery (9, 41). Our findings support this concept, indicating that the immune system in severe COVID-19 patients is shifting toward a more efficient and targeted response, with TCR-β (TRB) chains playing a central role in the recognition of SARS-CoV-2 and other potential co-infecting pathogens. In contrast, the decrease in TCR-β (TRB) usage reported in the acute phase of severe COVID-19 suggests immune dysfunction and T cell exhaustion, which are hallmarks of critical COVID-19 (14, 16, 42). Studies have demonstrated that during the acute phase, reduced TCR-β (TRB) chain usage is associated with impaired T cell functionality and a compromised ability to resolve the infection (43, 44). This aligns with findings from Giamarellos-Bourboulis et al. (45), who reported a reduction in TCR diversity and a decline in TCR-β (TRB) usage in severe COVID-19 patients, reflecting T cell exhaustion (45). The increased TCR-β (TRB) usage observed in our study at T3 therefore marks a recovery phase, where T cells are re-engaging with the virus to clear residual viral particles and establish long-term immunity, signifying a shift toward immune resolution. Also, the decreased prevalence of TCR-γ (TRG) chains at T3 suggests a shift from a more inflammatory, early immune response toward a more regulated immune state. TCR-γ (TRG) chains are often associated with the early stages of the immune response and are involved in the initial inflammatory and protective responses (46). The reduction in γ (TRG) chain usage at T3 may therefore reflect a transition from an acute inflammatory state toward a recovery phase, in line with the decreasing levels of pro-inflammatory cytokines we observed.

The preferential usage of V and J segments during the V(D)J recombination process plays a crucial role in shaping the TCR repertoire in response to infections. Our analysis revealed that the V and J segment profiles were broadly conserved across time points, with few differences observed between T1 and T3. Increased TRBV segments and decreased TRGV segment usage at T3 align with enhanced TCR-β (TRB) expansion and reduced TCR-γ (TRG) prevalence, reflecting an adaptive immune shift. These findings are in line with prior studies demonstrating selective V/J segment usage in viral infections, where certain segments are preferentially used to target specific pathogen-derived peptides (43, 47–49). The dynamic usage of V and J segments suggests a fine-tuned immune response, where TCRs evolve to target not only the viral antigens at early stages but also adapt to tackle any remaining or variant epitopes during the recovery phase.

The CDR3 region, known for its high variability, plays a critical role in antigen recognition and TCR repertoire diversity (50). Our findings of changes in the CDR3 motifs across time points offer further evidence of immune system adaptation. Notably, we observed a reduction in glycine residues in the central region of the CDR3 and the appearance of specific amino acids such as valine (V) and glutamine (Q) in δ-CDR3 (TRG) sequences at T3. These changes may reflect alterations in antigen-binding properties, potentially enhancing the TCR’s affinity for specific SARS-CoV-2 epitopes (33). Furthermore, the decrease in glycine residues, which are typically involved in flexible antigen binding, and the increase in other residues such as valine and glutamine suggest that the TCRs are evolving to better engage with persistent viral antigens (51). These changes in the CDR3 region likely contribute to the immune system’s ability to target viral peptides with increased specificity and efficiency as the disease progresses.

A shift toward immune regulation at T3 was further confirmed with the cytokine profiles. The significant decrease in pro-inflammatory cytokines, including IL-1β, IL-6, IP-10, and IL-1a, is a key indicator of the resolution phase of inflammation (52, 53). This reduction in inflammatory mediators correlates with a decrease in helper T-cell populations, suggesting that the immune system is transitioning from an active inflammatory state to a more regulated, recovery-oriented phase. Overall, the integration of TCR dynamics and cytokine profiles reveals a progression from acute inflammation at T1, driven by a narrow TCR repertoire and high pro-inflammatory cytokines, to a more adaptive phase at T2, where expanding TCR diversity correlates with moderated inflammation. By T3, the immune system achieves recovery, characterized by increased TCR diversity, reduced inflammatory and increased anti-inflammatory cytokines, indicating a balanced immune state aimed at resolving infection and establishing long-term immunity. Supporting this observation, we found that certain cytokines (e.g., IL-6 and IL-1β) showed opposing trends to TCR diversity indices at T3. While individual-level correlations at this time-point did not reach statistical significance, this pattern reinforces the longitudinal shift from inflammation to immune recovery. The reduction in helper T cells at T3 is also likely reflective of immune stabilization, allowing the immune system to establish a memory response and prepare for future exposures to the pathogen (54).

The increased diversity of epitopes recognized at T3, particularly those targeting key viral proteins such as the spike, ORF1ab, and ORF3 proteins, points to an adaptive immune response capable of recognizing a broader range of viral peptides. This expansion of epitope recognition suggests that, by T3, the immune system has mounted a more comprehensive defense against SARS-CoV-2, potentially clearing residual virus and preventing reinfection (12, 13, 55). Interestingly, we also observed the enrichment of epitopes from other pathogens, such as CMV, EBV, and *Plasmodium falciparum*, at T3. This cross-reactivity could be indicative of a heightened state of immune surveillance, where the immune system not only targets SARS-CoV-2 but also potentially prepares for secondary infections or the elimination of latent pathogens (56, 57). These epitopes, commonly represented in public databases due to widespread prior exposure and latent infection, may reflect the reactivation or recall of memory T cell populations during immune recovery. Rather than indicating active co-infection, their presence likely reflects a restored immune surveillance state or cross-reactivity. While these epitope annotations provide insight into potential antigen specificity, they are based on publicly available datasets and in silico predictions that may be biased toward well-characterized pathogens (e.g., CMV, EBV, Influenza). Additionally, these annotations do not account for individual HLA types and may miss novel or population-specific TCR–epitope interactions. As such, the findings should be interpreted as predictive and exploratory. The diversification of TCR responses in response to a wide array of antigens underscores the immune system’s capacity for a polyclonal response, which is crucial for effective pathogen clearance and immune memory formation.

**Figure 6:**
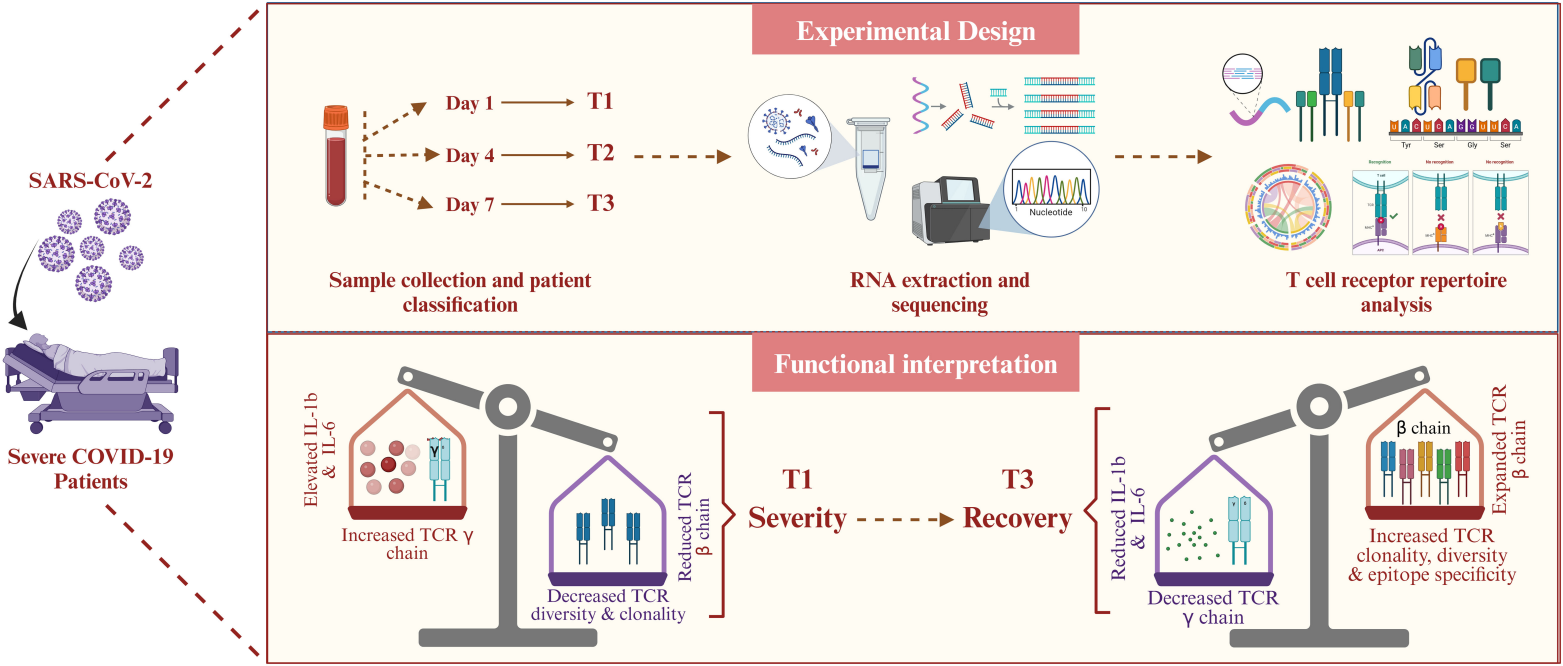
Summary of temporal TCR dynamics and immune recovery in severe COVID-19. The figure highlights key immunological shifts across time, including increased TCR clonotype diversity, TRB chain dominance, reduced inflammation, and predicted epitope diversification, capturing the transition from immune dysregulation to recovery in severe COVID-19.

In summary, this study provides a temporally resolved view of T cell immune recovery in severe COVID-19 patients. We observed a progressive expansion in TCR diversity and clonotype expansion, coupled with a shift from TCR-γ (TRG) to TCR-β (TRB) chain usage and preferential TRBV segment expression by Day 7. These repertoire changes coincided with a marked decline in pro-inflammatory cytokines (IL-1β, IL-6, IL-1α, IP-10), indicating a transition from acute inflammation to immune regulation. Additionally, computational epitope mapping revealed broadened antigen targeting and immune surveillance at T3, including predicted recognition of SARS-CoV-2 and latent pathogen epitopes. Together, these findings reflect a coordinated, polyclonal T cell response critical for resolving infection and restoring immune balance, offering insights into mechanisms of immune resilience with implications for future therapeutic and monitoring strategies (**Figure 6**).

### Limitations of the study

While this study offers meaningful insights into immune recovery in severe COVID-19, certain limitations should be considered when interpreting the findings. The identification of epitope-specific TCRs was based on publicly available databases (VDJdb and TCRex), which, although informative, may not fully capture rare or novel clonotypes, particularly those unique to underrepresented populations. Future incorporation of patient-specific HLA data and experimental validation methods would improve the specificity of these associations. Additionally, our reliance on bulk RNA sequencing, though suitable for assessing overall repertoire changes, limits the ability to assign clonotypes to particular T cell subsets or activation states. Single-cell sequencing based insights would enable a more precise view of how specific TCRs contribute to protective or dysregulated responses. Furthermore, the sparse annotation of TCR clonotypes to known SARS-CoV-2 epitopes across samples limited our ability to perform meaningful statistical correlations between cytokine profiles and antigen-specific T cell responses. This remains a critical area for future studies incorporating more comprehensive epitope prediction pipelines and paired single-cell cytokine and TCR profiling. Lastly, this cohort, derived from unvaccinated patients during the early pandemic phase, provides a consistent immunological baseline but may not reflect immune dynamics in vaccinated individuals or in infections caused by newer SARS-CoV-2 variants. Expanding such analyses to broader, more diverse populations will be essential for refining our understanding of T cell responses in evolving clinical contexts.

## Conclusion

Together, our findings offer compelling evidence of a dynamic TCR repertoire response to severe COVID-19 infection, transitioning from acute inflammation to a resolution phase. Enhanced TCR diversity and clonotype expansion at T3 highlight a polyclonal response critical for pathogen clearance. The preferential usage of TRBV segments and reduced TRGV prevalence reflect an adaptive immune shift. Changes in CDR3 motifs and cytokine profiles further indicate immune recovery, with increased specificity toward viral peptides. The enrichment of cross-reactive epitopes suggests heightened immune surveillance, underscoring the immune system’s adaptability and readiness for future challenges. In conclusion, this study offers a detailed perspective on analyzing the TCR dynamics in longitudinal ICU-admitted severe COVID-19 patients. This temporal focus allows for a deeper understanding of the shifts in TCR diversity, chain usage, and antigen specificity, directly linking these changes to immune recovery and modulation of inflammation. Our work adds a unique angle by integrating these findings with cytokine profiles, shedding light on the immune system’s progression from acute inflammation to recovery.

## Data Availability

The datasets presented in this study can be found online at the NCBI-SRA under the BioProject accession numbers PRJNA816679.
