# Supplementary material for: Temporal TCR dynamics and epitope diversity mark recovery in severe COVID-19 patients
Source: Front Immunol. 2025 Jul 10;16:1582949. doi: 10.3389/fimmu.2025.1582949 (PMC12286809; doi:10.3389/fimmu.2025.1582949)
Supplement: Supplementary file 1 [file DataSheet1.pdf]

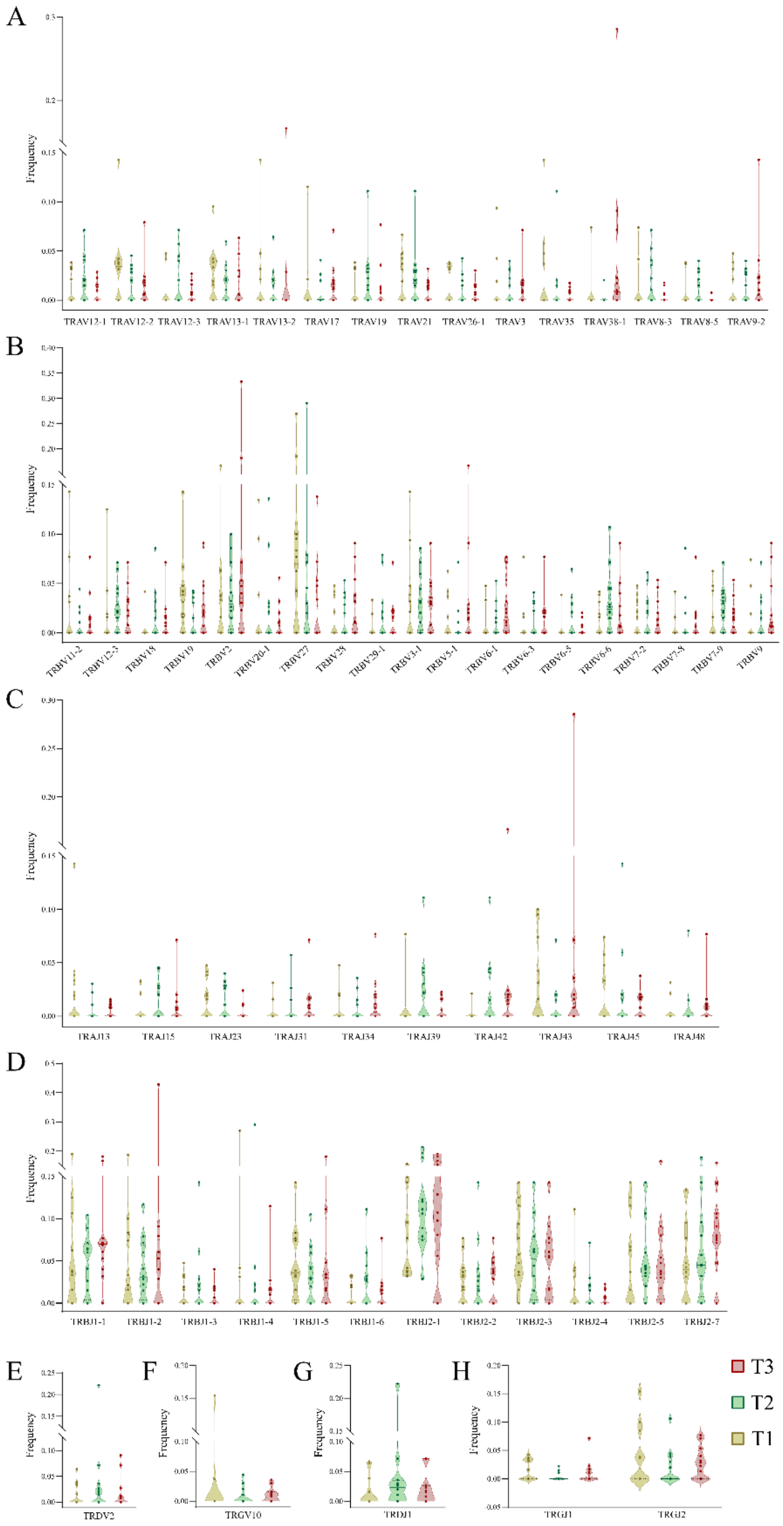

Figure S1. Comparison of the frequency of V and J gene segment usage between T1, T2, and T3 across TCR chains. Violin plots represents frequency of **(A)** V gene in TRA, **(B)** V gene in TRB, **(C)** J gene in TRA, **(D)** J gene in TRB, **(E)** V gene in TRD, **(F)** V gene in TRG, **(G)** J gene in TRD, and **(H)** J gene in TRG across T1, T2, and T3.

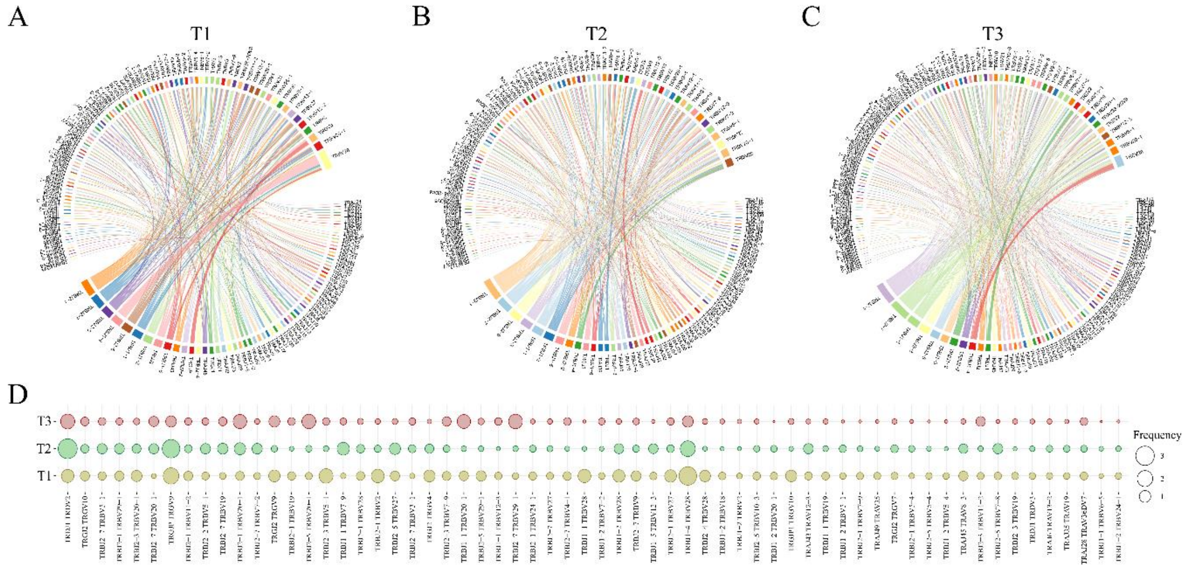

Figure S2. Frequency distribution of common VJ gene pairs within all TCR chains across T1, T2, and T3. Circos plots represents combination of VJ pairs across **(A)** T1, **(B)** T2, and **(C)** T3. **(D)** Bubble plot shows frequency distribution of topmost VJ pair within all TCR chains common across T1, T2, and T3.

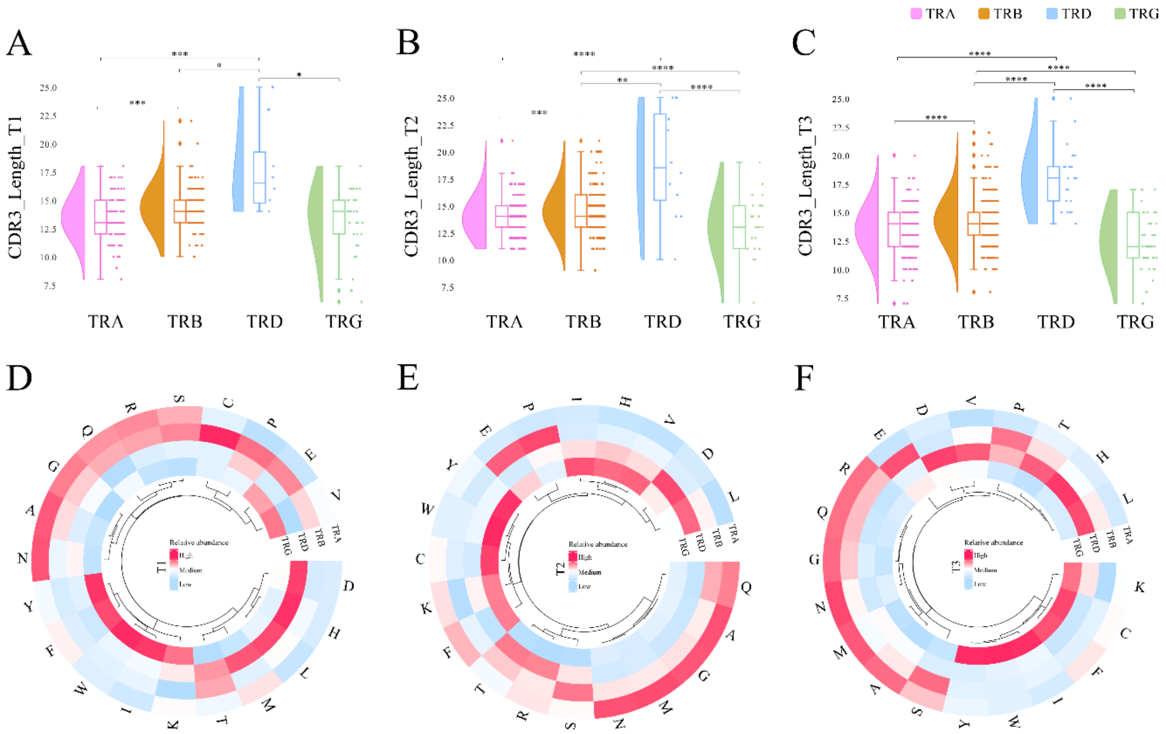

Figure S3. Distribution of CDR3 length and amino acid composition across all TCR chains between T1, T2, and T3. The raincloud plots represents CDR3 lengths across each TCR chains (TRA, TRB, TRD, and TRG) for **(A)** T1, **(B)** T2, and **(C)** T3. The circular heatmaps show the relative abundance of all 20 amino acids across each TCR chains (TRA, TRB, TRD, and TRG) for **(D)** T1, **(E)** T2, and **(F)** T3.
